# Roles of Trait Mindfulness in Behavioral Activation Mechanism for Patients With Major Depressive Disorder

**DOI:** 10.3389/fpsyg.2020.00845

**Published:** 2020-04-29

**Authors:** Koki Takagaki, Masaya Ito, Yoshitake Takebayashi, Shun Nakajima, Masaru Horikoshi

**Affiliations:** ^1^Health Service Center, Hiroshima University, Hiroshima, Japan; ^2^National Center for Cognitive Behavior Therapy and Research, National Center of Neurology and Psychiatry, Tokyo, Japan; ^3^Department of Health Risk Communication, School of Medicine, Fukushima Medical University, Fukushima, Japan

**Keywords:** behavioral activation, depression, trait mindfulness, avoidance, impairment

## Abstract

Behavioral activation and mindfulness have both been shown to engender improvement of functional impairment in patients with major depressive disorder. In behavioral activation, the practice of engaging with the direct experience of the present moment is central, especially when targeting avoidance. Consequently, mindfulness affects changes of avoidance in behavioral activation. This study was designed to assess exploratory relations among trait mindfulness, avoidance, and functional impairment in behavioral activation mechanism for depression. For 1042 participants with depression only or for depression with anxiety disorders, we used structural equation modeling to examine relations among trait mindfulness, avoidance, and functional impairment. Trait mindfulness non-reactivity, non-judging, and acting with awareness had a direct negative effect on avoidance. Trait mindfulness non-reactivity, trait non-judging, and trait acting with awareness had indirect negative effects on functional impairment. Results show that each trait mindfulness facet exhibited a distinct pattern of relations with avoidance and impairment.

## Introduction

Depression is a leading cause of absenteeism and reduced work productivity (Gilmour and Patten, 2007). Moreover, people with depression have an elevated risk of suicide (Pompili et al., 2012, 2013). Among the effective psychological treatments for depression, behavioral activation and mindfulness have been shown to engender improvement of functional impairment among patients with major depressive disorders (Dimidjian and Davis, 2009).

Psychotherapeutic approaches using mindfulness and behavioral activation, respectively, apparently target maladaptive psychological processes contributing to the maintenance of depression, such as engagement in avoidant behaviors (Dimidjian and Davis, 2009). Martell’s behavioral activation model (Martell et al., 2001) includes specific emphasis on the role of avoidance including avoidant behavior and rumination. In their model, which is consistent with traditional behavioral models, behavioral activation modifies a person’s environment through behavior change, which in turn increases access to positively reinforcing events and activities (Manos et al., 2010). In behavioral activation, the practice of engaging with the direct experience of the present moment is central, especially when targeting avoidance (Martell et al., 2010). In fact, a report of an earlier study described that mindfulness is linked to improvement of emotional processes (Fabio and Towey, 2018). Mindfulness is a mental state characterized by non-judgmental awareness of a present moment experience (Baer et al., 2004). Mindfulness can be conceptualized as a trait characteristic or suite of related characteristics, including the ability to observe and attend to experiences, the ability to describe those experiences, the ability to focus attention on the present moment, and the ability to adopt a non-judgmental attitude toward experiences (Baer et al., 2004). Theoretically, the practice of mindfulness can engender the occurrence of adaptive behavioral activation by canceling avoidant behavior through non-judgmental awareness (Kanter et al., 2009). Therefore, in a behavioral activation model for depression, mindfulness is an important factor to change avoidance to alternative behavior.

**FIGURE 1:**
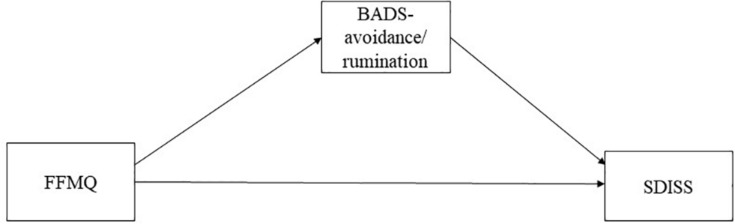
Hypothetical model in the simple model FFMQ, Five Facet Mindfulness Questionnaire; BADS, Behavioral Activation for Depression Scale; SDISS, Sheehan Disability Scale.

**FIGURE 2:**
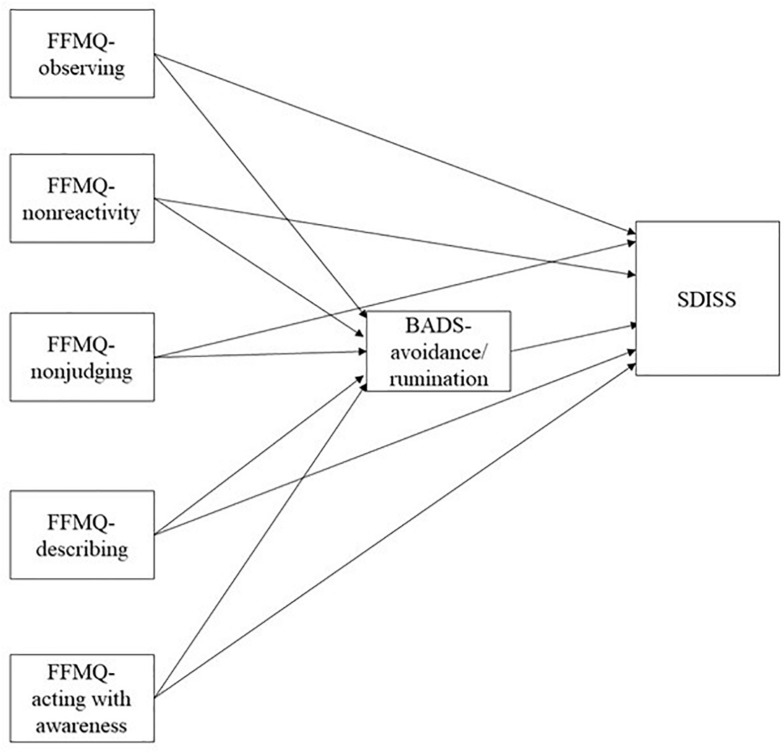
Hypothetical model in the detailed model. FFMQ, Five Facet Mindfulness Questionnaire; BADS, Behavioral Activation for Depression Scale; SDISS, Sheehan Disability Scale.

In meta-analysis conducted for an earlier study, trait mindfulness was found to be positively correlated with emotion regulation, mental health perceived life satisfaction, workplace functioning and professional outcome (Mesmer-Magnus et al., 2017). In addition, mindfulness is correlated negatively with avoidance (Baer et al., 2004), rumination (Desrosiers et al., 2013), and daytime impairment (Black et al., 2015). Takagaki et al. (2013b) described avoidance as positively correlated with functional impairment in a behavioral activation model. Therefore, based on results of several earlier studies (Baer et al., 2004; Desrosiers et al., 2013; Takagaki et al., 2013b; Black et al., 2015; Mesmer-Magnus et al., 2017), we hypothesized the following: trait mindfulness is negatively related to avoidance and impairment (Figure 1). Moreover, avoidance is positively correlated with impairment. Although earlier reports have described relations among those factors, trait mindfulness includes five abilities: observing, describing, acting with awareness, non-judging, and non-reactivity (Baer et al., 2006; Sugiura et al., 2012). Furthermore, earlier reports have described that characteristics of each trait mindfulness differ (Baer et al., 2006; Sugiura et al., 2012). Trait mindfulness except for observing was found to be negatively related with depressive symptoms, but Brown et al. (2015) found observation to be positively related with depressive symptoms. Trait mindfulness aside from observation and describing are negatively related to rumination (Dundas et al., 2013). Curtiss et al. (2017) described that observing was positively related to depression symptoms and that non-reactivity was positively related to reappraisal. Additionally, Teismann et al. (2016) demonstrated that increased awareness of present-moment experiences is associated with reduced avoidance and impairment. However, there has been lack of evidence among five trait mindfulness, avoidance, and impairment in the behavioral activation model of depression. Although we hypothesized that trait mindfulness is negatively related to avoidance and impairment based on earlier studies (Baer et al., 2004; Desrosiers et al., 2013; Black et al., 2015; Mesmer-Magnus et al., 2017), whether five trait mindfulness (observing, describing, acting with awareness, non-judging, and non-reactivity) is related to avoidance and functional impairment has not yet been investigated in behavioral activation for depression (Figure 2). Therefore, the primary purpose of this study was to assess the relation among five trait mindfulness, avoidance, and functional impairment in depression.

## Materials and Methods

### Participants and Procedures

This study is derived from a larger project for examining emotion and psychopathology. We obtained our data from results of a large web-based observational study (Ito et al., 2015). Details of the participants and procedures were presented in an earlier report. The sample of this study is same to previous study (Ito et al., 2015). In January and May 2014, 18-year-old or older panelists were recruited from those registered on Macromill Inc. Of the 1,095,443 registered panelists, 389,265 had been registered as “disease panelists.” Anonymous participants with depression were asked if they were currently diagnosed and were using medical services for treatment because Macromill’s operational definition of disease panelists had been reported by respondents 1 year before this study was conducted. We asked that are you currently diagnosed as having major depressive disorders (MDD) and being treated for the it in a medical setting? We posed the same questions for anxiety disorders (panic disorder, PD; social anxiety disorder, SAD; and obsessive–compulsive disorder, OCD). Participants were extracted randomly based on age, gender, and living area in each group. Furthermore, a report of an earlier study described that many participants’ characteristics were that they were able to use the internet and were young people with low income. Detailed descriptions of the participants were presented elsewhere (Ito et al., 2015). At that time, 1042 participants met the criteria for depression only or for depression with anxiety disorders. Of them, 406 participants met the criteria for only MDD. Also, 636 participants met the criteria for comorbid MDD and any anxiety disorder (127, MDD and PD; 95, MDD and SAD; 100, MDD and OCD; 51, MDD, PD, and SAD; 52, MDD, PD, and OCD; 55, MDD, SAD, and OCD; 156, MDD, PD, SAD, and OCD). We used data of 1042 participants for statistical analyses.

### Measures

#### Behavioral Activation for Depression Scale

The Japanese Behavioral Activation for Depression Scale (BADS), which comprises four subscales and 25 items. The four subscales include Activation (BADS-activation, BADS-AC), avoidance/Rumination (BADS-avoidance/rumination, BADS-AR), Work/School Impairment (BADS-work/school impairment, BADS-WS), and Social Impairment (BADS-social impairment, BADS-SI). The BADS-AC measures goal-directed activation and the completion of scheduled activities. The BADS-AR measures the avoidance of a negative aversive state, and engaging in rumination, rather than active problem solving. The BADS-WS measures the consequences of inactivity and passivity on work and school responsibilities. The BADS- SI measures similar social consequences and social isolation. BADS has good reliability and validity (Takagaki et al., 2013a). Cronbach alpha coefficients are adequate (BADS-A = 0.79, BADS-AR = 0.75, BADS-WS = 0.62, BADS-SI = 0.88, and BADS total = 0.78). This study specifically used BADS-AR. The avoidance/rumination subscale measures the avoidance of a negative aversive state and engagement in rumination rather than active problem-solving. We used a five-point Likert scale for each item to fit requirements of the larger web-based survey. A report of a study by Wakita et al. (2012) explained that descriptive statistics and reliability coefficients did not change significantly in accordance with response options. Confirmatory factor analysis results for the present sample showed acceptable fit to the data (CFI = 0.816, RMSEA = 0.098, SRMR = 0.083) in comparison to those of an earlier study (Kanter et al., 2007). Cronbach alpha coefficients were calculated for the four subscales (BADS-A = 0.82, BADS-AR = 0.84, BADS-WS = 0.78, BADS-SI = 0.91, and BADS total = 0.87).

#### Five Facet Mindfulness Questionnaire

The original Five Facet Mindfulness Questionnaire (FFMQ) comprises five subscales and 39 items, each of which is rated on a five-point Likert scale ranging from 1 (never or very rarely true) to 5 (very often or always true) (Baer et al., 2006). The five subscales include Observing (FFMQ-observing), Describing (FFMQ-describing), Acting with Awareness (FFMQ-acting with awareness), Non-judging (FFMQ-non-judging), and Non-reactivity (FFMQ-non-reactivity). The FFMQ-observing measure is applicable to internal or external environments. The FFMQ-describing measure can label the internal environment. The FFMQ-acting with awareness measure is applicable to the present moment. The FFMQ-non-judging measure does not allow to evaluate the internal environment. The FFMQ-non-reactivity measure is applicable to the internal environment without negative rumination. The Japanese version of the FFMQ has good reliability and validity (Sugiura et al., 2012). Cronbach alpha coefficients are adequate (FFMQ-observing = 0.79, FFMQ-describing = 0.75, FFMQ-acting with awareness = 0.62, FFMQ-non-judging = 0.88, FFMQ-non-reactivity = 0.78, and FFMQ-Total = 0.80). Cronbach alpha coefficients in this study were calculated for five subscales (FFMQ-observing = 0.81, FFMQ-describing = 0.84, FFMQ-acting with awareness = 0.85, FFMQ-non-judging = 0.85 and FFMQ-non-reactivity = 0.79. FFMQ-Total = 0. 78).

#### Sheehan Disability Scale

The Japanese Sheehan Disability Scale (SDISS), which comprises 1 factor and 3 items, has good reliability and validity (Yoshida et al., 2004). The SDISS is used to measure disability. Cronbach alpha coefficients are.84–0.87. The three items include impairment of work/school, social life, and family life/home responsibilities. We used a five-point Likert scale for each item to fit requirements of the larger web-based survey. Therefore, we conducted confirmatory factor analysis. Confirmatory factor analysis results for the present sample showed acceptable fit to data (CFI = 1.00, RMSEA = 0.00, SRMR = 0.00). The Cronbach alpha coefficient in this study was.92.

#### Patient Health Questionnaire (PHQ-9)

The Japanese version of the Patient Health Questionnaire (PHQ-9) comprises nine items. PHQ-9 is used to measure depressive symptoms. PHQ-9 has good reliability and validity (Muramatsu et al., 2007). The Cronbach alpha coefficient is 93. Cronbach alpha coefficients in this study was 92.

### Statistical Analysis

We conducted normality tests. However, not all factors had normality. We first report descriptive data. Next, we conducted analysis using Spearman correlation to examine relations among all factors. Results of an earlier study suggested that the bootstrap approach assumes that the sampling distributions of the total are normal when the underlying distribution is non-normal (Preacher and Hayes, 2008). Therefore, we examined relations among trait mindfulness, avoidance, and functional impairment using structural equation modeling (SEM) based on the maximum likelihood estimation method using bootstrapping.

We subsequently conducted an examination of the models including all variables. All observed variables were constructed using total scores. Several fit indices show how well the tested model accounts for the observed correlation structure of the data. The following indices were used for this study: chi-square test, comparative fit index (CFI), standardized root mean square residual (SRMR), and the root mean square error of approximation (RMSEA). The range of fit index values is 0–1 (Hooper et al., 2008). Reasonable fit is indicated by CFI values of 0.9 or more (Hooper et al., 2008). The RMSEA and SRMR lower limits are close to 0. Although the upper limit is expected to be less than 0.08, an RMSEA of 0.05–0.10 is regarded as an indication of fair fit (Hooper et al., 2008). The SRMR lower limit is close to 0, whereas the upper limit is expected to be less than 0.08 (Hooper et al., 2008). We used software for analyses (SPSS ver. 22.0; IBM Corp., Tokyo, Japan and Mplus ver. 7.4; Muthen & Muthen, Los Angeles, United States).

## Results

### Participant Characteristics and Correlations

Of participants, 1042 (549 women, 493 men; mean age 42.66 ± 9.22) reported that they are currently obtaining treatment for depression with anxiety or for depression only. Correlation analysis was applied to explore associations among all factors (Table 1).

**TABLE 1:** Descriptive data in depression.

	Mean (SD)	PHQ-9	BADS-AR	FFMO-observing	FFMQ-non-reactivity	FFMQ-non-judging	FFMQ-describing	FFMQ-acting with awareness
PHQ-9	14.52 (7.53)							
BADS-AR	13.48 (7.34)	0.57**						
FFMO- observing	21.57 (6.07)	0.26**	0.33**					
FFMQ-non-reactivity	17.10 (4.83)	−0.27**	−0.10**	−0.29**				
FFMQ-non-judging	24.13 (6.44)	−0.36**	−0.40**	−0.52**	−0.06*			
FFMQ-describing	20.87 (6.27)	−0.29**	−0.13**	−0.16**	0.44**	0.07*	–	–
FFMQ-acting with awareness	25.18 (6.23)	−0.45**	−0.44**	−0.41**	–0.06	0.48**	0.25**	
SDISS	5.88 (3.69)	0.68**	0.56**	0.27**	−0.22**	−0.31**	−0.19**	−0.34**

PHQ-9, Patient Health Questionnaire-9; BADS-AR, Behavioral Activation for Depression Scale-Avoidance/Rumination; The Five Facet Mindfulness Questionnaire-observing, FFMO- observing; FFMQ-non-reactivity, The Five Facet Mindfulness Questionnaire-non-reactivity; FFMQ-non-judging, The Five Facet Mindfulness Questionnaire-non-judging; FFMQ-describing, The Five Facet Mindfulness Questionnaire-describing; FFMQ-acting with awareness, The Five Facet Mindfulness Questionnaire-acting with awareness; SDISS, Sheehan Disability Scale. ** *p* > 0.01, * *p* > 0.05.

### Structural Equation Modeling

**FIGURE 3:**
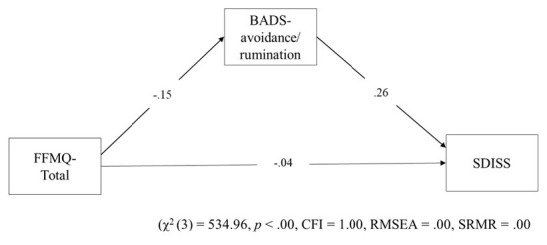
Hypothetical model in the simple model. FFMQ, Five Facet Mindfulness Questionnaire; BADS, Behavioral Activation for Depression Scale; SDISS, Sheehan Disability Scale; CFI, comparative fit index; RMSEA, root mean square error of approximation; SRMR, standardized root mean square residual.

**FIGURE 4:**
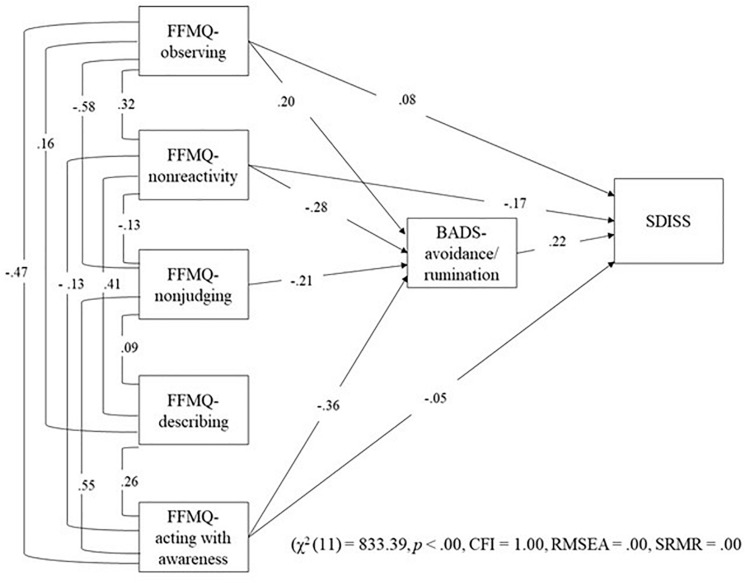
Results of SEMin the detailed model. FFMQ, Five Facet Mindfulness Questionnaire; BADS, Behavioral Activation for Depression Scale; SDISS, Sheehan Disability Scale; CFI, comparative fit index; RMSEA, root mean square error of approximation; SRMR, standardized root mean square residual.

We examined relations among trait mindfulness, avoidance, and functional impairment using SEM based on maximum likelihood estimation method using bootstrapping. Fitted index values suggested that the proposed model was valid (χ2 (3) = 534.96, *p* < 0.00, CFI = 1.00, RMSEA = 0.00, SRMR = 0.00; Figure 3). FFMQ-Total (standardized direct effect, −0.15; 95% confidence intervals, −0.18 to −0.13) had direct effects on BADS-AR. FFMQ-Total (standardized direct effect, −0.04; 95% confidence intervals, −0.06 to −0.03), and BADS-AR (standardized direct effect, 0.26; 95% confidence intervals, 0.23–0.28) had direct effects on SDISS. FFMQ-Total (standardized indirect effect, −0.04; 95% confidence intervals, −0.05 to −0.03) had indirect effects on SDISS. Next, we examined relations among five trait mindfulness, avoidance, and functional impairment using SEM based on with the maximum likelihood estimation method using bootstrapping. Fit index values suggested that the proposed model was valid (χ2 (11) = 833.39, p < 0.00, CFI = 1.00, RMSEA = 0.00, SRMR = 0.00; Figure 4). FFMQ-observing (standardized direct effect, 0.20; 95% confidence intervals, 0.12–0.28), FFMQ-non-reactivity (standardized direct effect, −0.28; 95% confidence intervals, −0.39 to −0.19), FFMQ-non-judging (standardized direct effect, −0.21; 95% confidence intervals, −0.28 to −0.13), and FFMQ-acting with awareness (standardized direct effect, −0.36; 95% confidence intervals, −0.44 to −0.27) had direct effects on BADS-AR. Moreover, FFMQ-observing (standardized direct effect, 0.08; 95% confidence intervals, 0.04 to 0.13), FFMQ-non-reactivity (standardized direct effect, −0.17; 95% confidence intervals, −0.21 to −0.13), FFMQ-acting with awareness (standardized direct effect, −0.05; 95% confidence intervals, −0.09 to −0.01), and BADS-AR (standardized direct effect,0.22; 95% confidence intervals, 0.19 to 0.26) had direct effects on SDISS. Moreover, results demonstrated that FFMQ-observing (standardized indirect effect, 0.04; 95% confidence intervals, 0.03 to 0.06), FFMQ-non-reactivity (standardized indirect effect, −0.06; 95% confidence intervals, −0.09 to −0.04), FFMQ-non-judging (standardized indirect effect, −0.05; 95% confidence intervals, −0.07 to −0.03), and FFMQ-acting with awareness (standardized indirect effect, −0.08; 95% confidence intervals, −0.10 to −0.06) had indirect effects on SDISS. FFMQ-describing was unrelated to BADS-AR and SDISS.

## Discussion

The primary purpose of this study was to assess the relation among five trait mindfulness, avoidance, and functional impairment in depression. The results of this study that trait mindfulness non-reactivity, non-judging, and acting with awareness had a direct negative effect on avoidance. Trait mindfulness non-reactivity, trait non-judging, and trait acting with awareness had indirect negative effects on functional impairment. The results found for SEM showed that each trait mindfulness facet showed a distinct pattern of relations with avoidant behavior and functional impairment. Results of this study demonstrate the differential pathways of mindfulness facets to functional impairment via avoidance. Behavioral activation targets avoidance as a primary difficulty posed by depression (Martell et al., 2001). Acting with awareness is related to efficient attention and cognitive control, whereas non-reactivity is related to metacognitive skills involving suspension of worry in the face of negative thoughts (Sugiura et al., 2012). Moreover, non-judging should be related to enhanced cognitive decentering (Sugiura et al., 2012). Ferster (1973) reported that many activities of depressed individuals are characterized by avoidance of aversive experiences. In behavioral activation therapy, treatment involves collaboration between the therapist and client to emphasize assessment of behavioral patterns that maintain depressive symptoms, and to increase activation of rewarding behaviors and effective problem solving (Martell et al., 2001). Avoidant behaviors function to alleviate participants’ distress in the short term, but they also increase depressive symptoms in the long term. Therefore, it is important to modify behaviors to achieve a long-term perspective. In enhancing the skills of acting with awareness, non-reactivity, and non-judging, results of our study indicate that individuals would be encouraged to modify negative cognitions related to specific experiences. They might be able to work on closely attending to specific behaviors, and might endeavor to foster an ability to activate helpful behaviors in the presence of negative affect. These skills would contribute to modification of avoidant behaviors to achieve a long-term perspective. A report of an earlier study described that the skills of acting with awareness, non-reactivity, and non-judging were improved by mindfulness-based interventions (van Emmerik et al., 2018). However, in this study, we were unable to examine the effectiveness that these skills affect modification of avoidant behaviors. Future studies must examine whether some exercises of mindfulness affect the skills of acting with awareness, non-reactivity, and non-judging in experimental research. The interesting results of this study show a deleterious effect of mindfulness observing impairment via avoidance. Observing was positively related to depression symptoms and anxiety symptoms (Brown et al., 2015; Curtiss et al., 2017). In addition, results of an earlier study suggest that observing appears to capture monitoring of experiences with reduced clarity, increased evaluation, and increased emotional distress (Sugiura et al., 2012). Avoidance is behavior that avoids negative aversive states. Therefore, increasing observation increases avoidance. Moreover, results of this study suggest that observation works to increase avoidance. Consequently, the implication is that one might decrease avoidance by decreasing observation.

This study has several limitations. The first limitation is participant selection. Participants were recruited via an internet survey, which can involve selection bias. To reduce the risk of bias, participants were recruited from various areas and age ranges. Because this was a web-based survey, it was limited to web users and biased to those who use the web frequently and to those who for whatever reason chose to complete a web-based survey. Even if one assumes that inclusion of people from multiple age groups and locations reduces this risk, it does not eliminate the risk and certainly does not allow generalization to non-web users and infrequent web users or to people who use the web but who choose not to fill out a form. Therefore, it is difficult to say how representative such a population might be of a clinical population or those coming to treatment for depressive illness. Moreover, many participants’ characteristics were that many study participants’ characteristics indicated they were young people with low income and ability to use the internet (Ito et al., 2015). In future research, it might be necessary to investigate this subject with only a clinical population or people seeking treatment for depressive illness. Secondly, we did not conduct a structured interview for assessing mental disorders. Additional studies must be conducted to generalize the results of this study using such a structured interview. Thirdly, we asked participants if they, at the time, were diagnosed as having MDD, PD, SAD, or OCD or were using medical services for treatment. However, we did not conduct structured interviews to assess mental disorders. Moreover, we did not check detailed information about somatic symptoms, medication, or psychotherapy. Also, although we did not check education or intelligence for this study, these factors are important to elucidate characteristics of the participants. Future studies should investigate these factors. Fourthly, we did not conduct an intervention in the earlier study. We conducted analyses by application of SEM to the relations among mindfulness, avoidance, and impairment in a process study. Therefore, interpretation of the possible causal relations must be done cautiously. Future studies should be conducted to investigate the causal relations using an appropriate experimental design. Fifth, only 406 participants met the criteria for only MDD, whereas 636 participants met the criteria for comorbid MDD and some anxiety disorder. The sample recruited for this study showed heterogeneous characteristics. Future studies should be conducted to analyze only the MDD sample. Finally, for this study, we hypothesized the following based on reports of some earlier studies: trait mindfulness is negatively related to avoidance and impairment. Furthermore, we examined relations among five trait mindfulness, avoidance, and functional impairment using structural equation modeling (SEM). However, it is possible that the discouragement and frustration resulting from functional impairment produce the psychological mindfulness impairments. Future studies must be conducted to examine relations among psychological mindfulness, avoidance, and functional impairment. In addition, this research is a cross-sectional study. Future studies must be conducted to examine causation of some factors by a longitudinal study or clinical trial. Despite these limitations, results of this study reveal robust relations of trait mindfulness in behavioral activation mechanisms. This report is the first describing a study examining relations between trait mindfulness, avoidance, and impairment. Each facet of trait mindfulness showed different relations with avoidant behavior.

For enhancing the skills of acting with awareness, non-reactivity, and non-judging, patients diagnosed with MDD or MDD and anxiety would be encouraged to modify negative cognitions related to specific experiences. They might improve their abilities to attend to specific behaviors and to activate helpful behaviors even in the presence of negative affect.

## Data Availability Statement

The datasets generated for this study will not be made publicly available as the authors do not have permission to share the data. Any questions regarding the data in this study should be directed to the corresponding author.

## Ethics Statement

The institutional review board at the National Center of Neurology and Psychiatry approved the ethical and scientific validity of this study (approval number: A2013-022). The survey was conducted on the Internet. Participants were asked to read the explanation of the study and provided informed consent by clicking the button of agreement before responding to questionnaires. All procedures performed in studies involving human participants were in accordance with the ethical standards of the institutional and/or national research committee.

## Author Contributions

KT, MI, YT, and SN conceived and designed the experiments. KT, MI, YT, SN, and MH contributed to the writing of the manuscript. All authors have approved the final manuscript.

## Conflict of Interest

The authors declare that the research was conducted in the absence of any commercial or financial relationships that could be construed as a potential conflict of interest.
